# The interplay between spiritual care practice, perception, and competency among critical care nurses: structural equation modeling study

**DOI:** 10.1186/s12912-026-05176-9

**Published:** 2026-08-11

**Authors:** Eman Arafa Hassan, Maysa Abdalla Ali Elbiaa, Shimmaa Mohamed Elsayed

**Affiliations:** 1 Critical Care and Emergency Nursing, Critical Care and Emergency Nursing Department, Faculty of Nursing, Alexandria University, Alexandria, Egypt; 2 Critical Care and Emergency Nursing, Critical Care and Emergency Nursing Department, Faculty of Nursing, Matrouh University, Matrouh, Egypt; 3 Critical Care and Emergency Nursing, Critical Care and Emergency Nursing Department, Faculty of Nursing, Damanhour University, El Beheira, Egypt

**Keywords:** Critical care nursing, Competence, Perception, Spirituality, Structural equation modeling

## Abstract

**Background:**

Spiritual care is a vital yet often overlooked component of holistic nursing, particularly in high-stress environments similar intensive care units. While critical care nurses’ spiritual perceptions and practices have been investigated, the mediating role of competency and the influence of demographic factors remain understudied.

**Aim:**

This study aimed to investigate relationships among spiritual perception, competency, and practice in critical care nurses, focusing on how competency mediates these relationships and the potential moderating role of demographic variables.

**Methods:**

A multicenter cross-sectional study was conducted with a convenience sample of 312 critical care nurses across three hospitals in Egypt. Data were collected from 14 intensive care units between February and March 2024 using an online questionnaire that included the Spiritual Care Practice Questionnaire, the Perception of Spirituality and Spiritual Care Rating Scale, and the Spiritual Care Competency Scale. Structural equation modeling was employed to analyze mediation and moderator variables.

**Results:**

A total of 312 nurses participated in the study. Spiritual competency significantly mediated the relationship between spiritual perception and Spiritual Care Practice (unstandardized indirect effect = 0.302, *p* < 0.001; standardized β = 0.357). Structural relationships were evaluated based on individual path coefficients rather than the global model fit. Additionally, age significantly moderated the paths from spiritual perception to practice and from spiritual competency to practice; ICU type significantly moderated the paths from spiritual perception to practice, from spiritual perception to competency, and from competency to practice; gender did not significantly moderate any path; and education level significantly moderated the paths from spiritual perception to practice, from spiritual perception to competency, and from competency to practice. Years of ICU experience did not show a significant moderating role.

**Conclusion:**

Spiritual care competency was associated with the relationship between perception of spirituality and spiritual care and its practice among nurses. Specific demographic characteristics demonstrated significant interaction effects on selected pathways. Age and ICU type showed significant interaction effects on several structural pathways, whereas years of ICU experience did not demonstrate significant interaction effects, underscoring the need for tailored approaches to spiritual care training and practice. Integrating spiritual care competency into training programs for critical care nurses, while considering demographic factors, may enhance holistic care in intensive care units.

**Clinical trial:**

Not applicable.

## Introduction

In critical care, holistic care that includes physical and spiritual needs is essential. Spiritual care, involving healthcare providers’ practices and perceptions of patients’ spiritual needs, is now a vital part of patient-centered care [1]. Despite this growing recognition, the mechanisms through which spiritual care perceptions and competencies influence the actual practice of spiritual care remain underexplored, particularly in high-stakes settings such as intensive care units (ICUs) [2, 3]. This study examines the interrelated factors of Spiritual Care Practice, perception, and competencies to clarify how they affect nurses’ ability to provide meaningful spiritual care in the demanding ICU environment.

This study is grounded in an integrated theoretical framework combining Jean Watson’s Theory of Human Caring and Albert Bandura’s Self-Efficacy Theory in a complementary manner [4, 5]. Watson’s theory emphasizes the significance of spirituality in nursing, advocating a holistic approach that nurtures both the patient’s body and spirit. Within this framework, spiritual care is not merely an adjunct to medical treatment but a fundamental aspect of the healing process. Watson’s emphasis on the relational aspects of care provides a foundation for understanding how nurses’ spiritual perceptions shape their caregiving behaviors [4]. Complementing this, Bandura’s Self-Efficacy Theory provides insight into the role of perceived competency in the execution of spiritual care [5]. According to Bandura, self-efficacy, the belief in one’s ability to perform specific tasks, is associated with behavior. In this context, spiritual care competency was modeled as a mediator because nurses’ perceptions of spirituality and spiritual care may be associated with their readiness, knowledge, skills, self-confidence, and ability to provide such care, which, in turn, may be associated with Spiritual Care Practice. Nurses with high self-efficacy in spiritual care are more likely to translate their perceptions and beliefs into concrete practices. This study uses an integrated framework that combines Watson’s Theory of Human Caring with Bandura’s Social Cognitive Theory to explain how ethical values translate into professional practice. While Watson’s theory offers the ontological foundation, emphasizing spiritual awareness and the capacity to find meaning as essential conditions for caring, Bandura’s theory introduces the behavioral mechanism of “self-efficacy,” which functions as the driving force that transforms this internal awareness into external action. Accordingly, the study proposes a practical alignment that links the “philosophy of caring” (why we care) with the “mechanisms of implementation” (how we apply it), where perceived self-efficacy acts as a mediator linking nurses’ awareness of their spiritual selves to their actual spiritual practices, while considering contextual and demographic factors that influence this complex human interaction. [5]. These theories suggest caring consciousness promotes perceived spiritual care competency, leading to spiritual care in practice via a cognitive–motivational mechanism. This process transforms caring awareness into action and transpersonal relationships, fostering sensitivity to patients’ spiritual needs and underpinning spiritual perception and care practice [4, 5]. This study views competency as a mediator translating internal cognitive and emotional states into clinical performance. Based on social cognitive theory, self-efficacy affects behavior by shaping skill development and use; therefore, competency is the key mechanism through which these internal beliefs manifest in practice.

Previous research highlights the importance of spiritual care in nursing but often treats its components separately. Studies show nurses’ spiritual perceptions relate to their willingness to provide spiritual care [6, 7]. Other research has focused on developing spiritual care competencies through education and training [8, 9]. However, few studies have examined how these dimensions interact within the broader context of nursing, and few studies have examined these constructs specifically in critical care nursing [10, 11].

While existing literature highlights the importance of spiritual care, there is a paucity of research that integrates spiritual perception, competency, and practice into a cohesive model [11]. The interaction between these variables, particularly within the demanding environment of the ICU, has not been sufficiently explored [1, 12]. Additionally, the use of advanced statistical methods, such as Structural Equation Modeling (SEM), to unravel the complex relationships among these constructs is notably limited in the current CCN literature in Egypt, particularly in a multicenter context [13, 14]. Furthermore, to the best of our knowledge, no prior study has simultaneously examined self-efficacy as a mediating variable alongside demographic factors as moderators within a single SEM framework in this population. This study examines Egyptian critical care nurses using a multicenter and SEM approach. Unlike previous cross-sectional studies that only report bivariate associations, this study tests direct, mediating, and moderating relationships in a single model, offering a clearer explanation of how caring constructs influence professional practice. This study aims to address this gap by employing SEM to investigate the interplay among spiritual perception, competence, and practice among critical care nurses. Additionally, assess the moderating role of nurses’ demographic characteristics on these relationships.

**Research hypothesis**

H1: There is a positive relationship between spiritual perception and Spiritual Care Practice among critical care nurses.

H2: Spiritual competency mediates the relationship between spiritual perception and Spiritual Care Practice among critical care nurses.

H3. Nurses’ age, gender, ICU type, level of education, and years of ICU experience moderate the direct effects of spiritual perception on Spiritual Care Practice.

H4. These demographic variables may also moderate the associations between spiritual perception and spiritual care competency, and between spiritual care competency and Spiritual Care Practice.

## Methods

### Design

This study employed an observational, multicenter, cross-sectional design using a self-administered online questionnaire. The study was conducted and reported in accordance with the Strengthening the Reporting of Observational Studies in Epidemiology (STROBE) guidelines [15]. 

### Settings

Data for this study were collected from fourteen ICUs across three hospitals in Egypt. These included four general adult ICUs, three medical ICUs, three surgical ICUs, one trauma ICU, two cardiac ICUs, and one neurological ICU.

### Participants

The target population for this study comprised nurses working in intensive care units. A convenience sampling technique was employed. Inclusion criteria were registered nurses who provided direct patient care and had at least 6 months of experience in a critical care setting. Nurses on any type of leave were excluded.

To determine the required sample size, we considered the complexity of the structural equation model, which included three higher-order latent constructs (Spiritual Perception, Spiritual Competency, and Spiritual Care Practice) measured by 11 observed indicators (subscales). According to established guidelines for SEM, a sample size exceeding 200–400 participants is generally adequate for models of moderate complexity when using maximum likelihood estimation, with a recommended minimum of 10–20 observations per observed variable. Our final sample of 312 nurses comfortably exceeded these recommendations, providing sufficient statistical power and parameter stability.

As a supplementary conservative estimate, a power analysis was conducted using G*Power (version 3.1.9.7) for an F-test in a multiple regression framework (fixed model, R² deviation from zero). Assuming a moderate effect size (f² = 0.15), α = 0.05, and power = 0.95, the minimum required sample size was 184. The achieved sample of 312 participants therefore exceeded both the SEM-specific guidelines and the supplementary power calculation.

### Data collection

Data for this study were collected between February and March 2024 via an online questionnaire comprising four sections. All sections were administered in their original English versions. Prior to the main study, content validity for use with Egyptian ICU nurses was evaluated by a panel of five Egyptian critical care nursing experts who confirmed the appropriateness and clarity of the items. A pilot study was conducted with 30 critical care nurses (not included in the final sample) to assess clarity, readability, completion time (approximately 10–12 min), and technical functionality. Minor formatting adjustments were made based on pilot feedback.

The first section included sociodemographic and work-related characteristics of nurses, such as age, gender, academic qualifications, years of ICU experience, and the type of ICU in which they were employed.

The second section was the spiritual care practice questionnaire developed by Gallison et al. (2013) [16]. This questionnaire includes two subscales: spiritual support and barriers to providing spiritual care. The spiritual support subscale comprises three items rated on a 5-point Likert scale ranging from 1 (very seldom) to 5 (very often). This subscale demonstrated good internal consistency (Cronbach’s alpha = 0.87). The barriers to providing spiritual care subscale consists of eight items. Responses are binary, with options for agree (scored as 2) and disagree (scored as 1). The internal consistency of this subscale was moderate (Cronbach’s alpha = 0.64) as reported in Gallison et al.‘s (2013) study [16]. The spiritual care practice construct encompasses both enacted spiritual support behaviors and perceived barriers to spiritual care. This operationalization reflects nurses’ active engagement with spiritual care alongside their awareness of contextual obstacles.

In this study, higher scores on the Barriers to Providing Spiritual Care subscale were intentionally retained as indicating greater perceived barriers and were not reverse-coded. Both Spiritual Support and Barriers to Providing Spiritual Care were modeled as reflective indicators of a higher-order latent construct labeled Spiritual Care Practice. This operationalization reflects the view that Spiritual Care Practice encompasses not only the frequency of supportive behaviors but also a heightened professional awareness of systemic and organizational obstacles that arise when nurses actively attempt to provide spiritual care. For descriptive analyses, subscale scores were combined to yield an overall spiritual care practice score (range 16–30, higher scores indicating greater practice), with the Spiritual Support subscale summarized as a sum of Likert-type items and the Barriers subscale summarized as a summed binary score. In the SEM, however, the two subscales were specified as separate reflective indicators of the higher-order Spiritual Care Practice latent construct, rather than merging all items into a single composite. This specification allowed the model to accommodate the different response formats while focusing on the shared variance between the support and barrier dimensions of practice.

Given the conceptual distinction between enacted spiritual support and perceived barriers, the Spiritual Care Practice construct should be interpreted as representing spiritual care engagement together with contextual barrier awareness rather than behavioral practice alone. The inclusion of the barriers indicator reflects nurses’ recognition of organizational and clinical constraints encountered during spiritual care delivery. Therefore, findings related to this construct should be interpreted with consideration of this broader operational definition.

The Spiritual Support subscale consists of three items rated on a 5-point Likert scale. For descriptive analyses, item scores were summed to generate a total score ranging from 3 to 15, with higher scores indicating more frequent provision of spiritual support. The same summed score was used as the observed indicator in the SEM analysis. The Barriers to Providing Spiritual Care subscale (8 items, binary agree/disagree) was scored as the sum (range 8–16; higher scores = greater perceived barriers), without reverse coding. Both subscales were entered into the SEM as reflective indicators of a higher-order latent construct labelled “Spiritual Care Practice.” The positive loading of the Barriers subscale on this latent variable indicates that nurses who engage more actively in spiritual care also tend to perceive more barriers, which is conceptually plausible and consistent with prior literature.

The third section of the questionnaire focused on spiritual perception and was measured using the Spirituality and Spiritual Care Rating Scale. This scale was developed by McSherry et al. (2002) [17]. This scale includes 17 items distributed across four subscales: spirituality (5 items), spiritual care (5 items), religiosity (3 items), and personalized care (4 items). Responses were recorded on a 5-point Likert scale ranging from 1 (strongly disagree) to 5 (strongly agree). The scale demonstrated good internal consistency, with an overall Cronbach’s alpha of 0.74 as reported by McSherry et al. (2002) [17]. 

The fourth and final section was the Spiritual Care Competency Scale, developed by van Leeuwen et al. (2009) [18]. This scale consists of 27 items across six subscales: assessment and implementation of spiritual care (6 items), professionalization and improving quality of spiritual care (6 items), personal support and patient counseling (6 items), referral to professionals (3 items), attitude towards patient spirituality (4 items), and communication (2 items). Responses were rated on a 5-point Likert scale ranging from 1 (completely disagree) to 5 (fully agree). All subscales demonstrated acceptable internal consistency, with Cronbach’s alpha values exceeding 0.7 in van Leeuwen et al.‘s (2009) study [18]. 

The reliability of all scales was retested in the current sample to assess consistency within the Egyptian cultural context and is reported in Table 2.

The latent construct “Spiritual Care Practice " herein encompasses both enacted spiritual support behaviors and professional awareness of systemic and organizational obstacles that arise when nurses actively attempt to provide spiritual care. Readers should interpret this construct as a composite index of practice engagement and contextual obstacle recognition, rather than purely as a measure of behavioral frequency. Both Spiritual Support and Barriers to Providing Spiritual Care were modeled as reflective indicators of a higher-order latent construct labeled Spiritual Care Practice. The positive loading of the Barriers subscale on this latent variable indicates that nurses who engage more actively in spiritual care also tend to perceive more barriers, which is conceptually plausible and consistent with prior literature showing that experienced spiritual care providers often report more barriers precisely because they attempt to overcome them (16, 29).

The questionnaire was created using Google Forms and distributed via dedicated WhatsApp groups for each participating ICU. Weekly reminder messages were sent, and a designated nurse in each unit facilitated distribution. To prevent duplicate responses, Google Forms was configured with the “limit to one response” option using browser cookies, so participants who had already submitted the questionnaire received an automatic message stating that they had already responded. No Google account login, IP addresses, emails, or personal identifiers were collected. All questionnaire items were mandatory; participants could not submit the form until all items were completed. Missing data were minimal (< 2%) and handled via listwise deletion in the SEM analysis. A designated nurse from each ICU facilitated the distribution of questionnaires and the reminder process.

### Ethical considerations

Participant involvement in this study was voluntary, and electronic informed consent was obtained from all nurses via the first page of the Google Forms questionnaire before they could access the survey items. The research ethics committee of the Faculty of Nursing, Alexandria University (IRB: IRB00013620, reference number: 2022-9-69) approved the study. To safeguard participant confidentiality, all data were anonymized, and the principal investigator maintained them in a secure storage location. All procedures followed the tenets of the Declaration of Helsinki.

### Data analysis

Data from this study were analyzed using JASP (version 0.19.0.0) and R (version 4.3.3). The Kolmogorov–Smirnov test was used to assess normality. Mean and standard deviation were used for continuous variables, while frequencies and percentages were reported for categorical variables. The internal consistency of the scales was assessed using Cronbach’s alpha. Weights and loadings of the study constructs were analyzed to present the relative importance of each indicator in predicting the latent construct.

The SEM analysis was conducted using the lavaan package to examine both mediation and moderator variables. The fit of the SEM model was assessed through various goodness-of-fit indices. A non-significant Chi-Square (χ²) value indicates a good fit between the model and the observed data. The Comparative Fit Index (CFI) values above 0.90 suggest an acceptable fit, while values above 0.95 indicate a good fit. Similarly, the Tucker-Lewis Index (TLI) considers values above 0.90 as acceptable. Root Mean Square Error of Approximation (RMSEA) values below 0.08 are considered adequate, with values below 0.05 indicating a good fit. Lastly, the Standardized Root Mean Square Residual (SRMR) values below 0.08 also suggest a good fit [19]. Additionally, standardized path coefficients were calculated to determine the strength of the relationships between variables.

Categorical demographic variables (gender, ICU type, and education level) were dummy-coded as indicator variables. Gender was coded as Male = 0 (reference) and Female = 1. Education level was dummy coded with bachelor’s as the reference category (Technical = 1, Master’s = 1). ICU type was dummy coded with General ICU = 0 (reference), Medical ICU = 1, Surgical ICU = 1, and Trauma ICU = 1. Coronary ICU and Neurological ICU were excluded due to small sample sizes (*n* < 10).

## Results

A total of 312 critical care nurses participated in the study, representing an 83% response rate among the target population.

### Sociodemographic and work-related characteristics of critical care nurses

The mean age of nurses was 31.85 ± 7.63 years. The sample was predominantly female (61.54%), with a high proportion holding a bachelor’s degree (54.81%); 39.74% held a technical degree, and 5.45% held a master’s degree. Nurses were distributed across various ICU types, with the majority working in General ICUs (45.83%), followed by Medical (22.44%) and Surgical (22.44%) ICUs. Smaller percentages of nurses worked in specialized units, including Trauma ICUs (5.13%), Coronary ICUs (2.24%), and Neurological ICUs (1.92%). The mean ICU years of experience was 6.16 ± 4.14 years (Table 1).

**Table 1:** Sociodemographic and work-related characteristics of critical care nurses (*n* = 312)

Variable	*N* (%) or mean (SD)
Age	31.85 (7.63)
Gender	
Female	192 (61.54%)
Male	120 (38.46%)
Educational level	
Technical degree	124 (39.74%)
Bachelor’s degree	171 (54.81%)
Master’s degree	17 (5.45%)
Type of ICU	
General ICU	143 (45.83%)
Medical ICU	70 (22.44%)
Surgical ICU	70 (22.44%)
Trauma ICU	16 (5.13%)
Coronary ICU	7 (2.24%)
Neurological ICU	6 (1.92%)
ICU Experience	6.16 (4.14)

### Descriptive statistics and reliability of scales and constructs

Table 2 highlights the key descriptive statistics and reliability for the primary scales used in this study: Spiritual Care Practice, spiritual care perception, and spiritual care competency. The Spiritual Care Practice scale, assessed using 11 items, yielded a mean score of 22.14 ± 2.25 and Cronbach’s alpha of 0.78, indicating acceptable internal consistency. The Spiritual Support subscale (3 items, 5-point Likert) was scored as a sum for descriptive presentation (range 3–15; higher scores indicate more frequent spiritual support). The Barriers to Providing Spiritual Care subscale (8 items, binary agree/disagree) was scored as a sum (range 8–16; higher scores = greater perceived barriers), without reverse coding. The spiritual care perception scale, comprising 17 items, had a mean score of 62.18 ± 6.69 and a Cronbach’s alpha of 0.88, indicating strong internal consistency. Lastly, the spiritual care competency scale, comprising 27 items, had a mean score of 95.58 ± 8.85 and a Cronbach’s alpha of 0.84, indicating good reliability.

**Table 2:** Descriptive statistics and reliability of scales and constructs

Construct and indicators	Number of items	Minimum	Maximum	Mean	SD	Cronbach’s Alpha
Spiritual Support	3	7.00	15.00	10.90	1.80	0.73
Barriers to Providing Spiritual Care	8	8.00	16.00	11.24	1.36	0.82
**Spiritual Care Practice (Total)**	**11**	**16.00**	**30.00**	**22.14**	**2.25**	**0.78**
Spirituality Perception	5	15.00	25.00	19.27	2.69	0.86
Perception of Spiritual Care (subscale)	5	14.00	25.00	18.41	2.67	0.90
Religiosity Perception	3	8.00	14.00	10.43	1.53	0.89
Personalized Care Perception	4	12.00	17.00	14.07	1.74	0.85
**Spiritual Care Perception (Total)**	**17**	**52.00**	**81.00**	**62.18**	**6.69**	**0.88**
Assessment and Implementation of Spiritual Care	6	16.00	30.00	22.36	4.08	0.82
Professionalization and Improving Quality	6	12.00	24.00	20.88	3.01	0.77
Personal Support and Patient Counseling	6	17.00	25.00	20.77	2.53	0.93
Referral to Professionals	3	8.00	14.00	10.75	1.45	0.77
Attitude Towards Patient Spirituality	4	9.00	18.00	13.89	1.92	0.91
Communication	2	5.00	8.00	6.93	0.92	0.86
**Spiritual Care Competency (Total)**	**27**	**76.00**	**108.00**	**95.58**	**8.85**	**0.84**

### Measurement model (CFA)

Prior to testing the structural model, a confirmatory factor analysis (CFA) was conducted to evaluate the measurement model. The three higher-order latent constructs (Spiritual Perception, Spiritual Competency, and Spiritual Care Practice) were specified with their respective subscales as reflective indicators.

The measurement model demonstrated acceptable fit: χ²(186) = 478.62, *p* < 0.001, CFI = 0.942, TLI = 0.935, RMSEA = 0.067 (90% CI: 0.060–0.074), SRMR = 0.058. All standardized factor loadings were statistically significant and ranged from 0.782 to 0.983. Although several loadings exceeded 0.95, multicollinearity diagnostics showed variance inflation factors (VIF) below 3.0. Discriminant validity was supported using both the Fornell–Larcker criterion (the average variance extracted for each construct exceeded the squared correlation between that construct and any other construct) and heterotrait–monotrait (HTMT) ratios (< 0.85).

Composite reliability (CR) and average variance extracted (AVE) were calculated to further assess convergent validity and construct distinctiveness. The values were as follows: Spiritual Care Practice (CR = 0.89, AVE = 0.79), Spiritual Perception (CR = 0.96, AVE = 0.87), and Spiritual Competency (CR = 0.96, AVE = 0.82). Although these statistics indicate strong convergent validity, several standardized loadings exceeded 0.95 (e.g., Personal Support and Patient Counseling = 0.983; Professionalization and Improving Quality = 0.976). Such high values suggest considerable conceptual overlap or redundancy among certain subdimensions of spiritual perception and spiritual competency. This pattern indicates that specific facets may be highly interrelated in the ICU context, and future research should consider whether a more parsimonious factor structure or revised item content could better differentiate these domains.

Factor weights (also referred to as relative importance weights) represent the proportionate contribution of each indicator to the formation of its respective latent construct. These weights were estimated using the lavaan package and are presented in Table 3 along with standardized loadings.

### Factor weights and loadings of constructs and indicators

Table 3 summarizes the factor weights and loadings for the constructs of Spiritual Care Practice, spiritual care perception, and competency. In the Spiritual Care Practice domain, barriers to providing spiritual care showed the highest weight (0.519) and the strongest loading (0.929), while spiritual support had a notable weight (0.418) and substantial loading (0.852). In the spiritual perception construct, the Perception of Spiritual Care subscale had the highest loading (0.955) and weight (0.252), followed by personalized spiritual care perception (loading 0.939; weight 0.250), religiosity perception (loading 0.926; weight 0.229), and spirituality perception (loading 0.902; weight 0.137). Within spiritual competency, personal support and patient counseling had the highest loading (0.983), followed by professionalization and improving quality of spiritual care (loading 0.976). Communication contributed the highest weight (0.364) with a strong loading (0.911), while referral to professionals (loading 0.818; weight 0.185) and attitude toward patient spirituality (loading 0.782; weight 0.142) also made meaningful contributions.

**Table 3:** Factor weights and loadings of constructs and indicators for spiritual care practice, perception, and competency

Construct	Indicator	Unstandardized Estimate	SE	z-value	*p*-value	Standardized Loading (β)	Weight Estimate
Spiritual Care Practice	Spiritual Support	1.000 (fixed)	—	—	—	0.852	0.418
	Barriers to Providing Spiritual Care	1.820	0.145	12.55	< 0.001	0.929	0.519
Spiritual Perception	Spirituality Perception	1.000 (fixed)	—	—	—	0.902	0.137
	Spiritual Care Perception	1.450	0.112	12.95	< 0.001	0.955	0.252
	Religiosity Perception	1.280	0.098	13.06	< 0.001	0.926	0.229
	Personalized Care Perception	1.320	0.105	12.57	< 0.001	0.939	0.250
Spiritual Competency	Assessment and Implementation of Spiritual Care	1.000 (fixed)	—	—	—	0.929	0.170
	Professionalization and Improving Quality	1.150	0.085	13.53	< 0.001	0.976	0.177
	Personal Support and Patient Counseling	1.220	0.092	13.26	< 0.001	0.983	0.179
	Referral to Professionals	0.950	0.078	12.18	< 0.001	0.818	0.185
	Attitude Towards Patient Spirituality	0.880	0.072	12.22	< 0.001	0.782	0.142
	Communication	1.080	0.088	12.27	< 0.001	0.911	0.364

### Goodness-of-fit of the structural equation model

The hypothesized structural equation model demonstrated an acceptable to good fit with the data as indicated in Table 4. The goodness-of-fit indices (χ² = 478.62, df = 186, *p* < 0.001; CFI = 0.942; TLI = 0.935; RMSEA = 0.067; SRMR = 0.058) reflect the adequacy of the measurement model (CFA) only. Since the structural portion of the model is just-identified, these indices cannot be interpreted as empirical validation of the hypothesized directional pathways.

Because the structural portion of the model is just-identified (three latent variables with all directed paths and residual variances freely estimated), the overall SEM fit statistics are identical to those of the CFA. Consequently, the measurement model dictates the fit of the overall SEM, and the addition of the structural regression paths does not alter the χ², CFI, TLI, RMSEA, or SRMR values.

Because the structural portion of the proposed model was just-identified, the reported global fit indices primarily represent the fit of the measurement model rather than the validity of the hypothesized structural relationships. Therefore, these indices should not be interpreted as confirming the proposed directional pathways. The structural relationships were evaluated based on the magnitude, statistical significance, and confidence intervals of individual path coefficients.

**Table 4:** Goodness-of-fit indices for the final structural equation model

Fit Index	Value	90% CI
χ² (df, p-value)	478.62 (186, < 0.001)	—
χ²/df	2.57	—
CFI	0.942	—
TLI	0.935	—
RMSEA	0.067	0.060–0.074
SRMR	0.058	—

### Explained variance in the endogenous variables

The structural equation model accounted for a substantial proportion of variance in the key endogenous latent variables as indicated in Table 5. Specifically, spiritual perception and other predictors explained 57.5% of the variance in spiritual competency (R² = 0.575). For Spiritual Care Practice, the model (including direct effects from spiritual perception and indirect via spiritual competency, plus moderation terms) explained 42.3% of the variance (R² = 0.423).

**Table 5:** Explained variance (R²) for endogenous latent variables

Endogenous Variable	*R*² (Squared Multiple Correlation)
Spiritual Competency	0.575
Spiritual Care Practice	0.423

### Mediation analysis of spiritual competency on the relationship between spiritual perception and practice

**Fig. 1:**
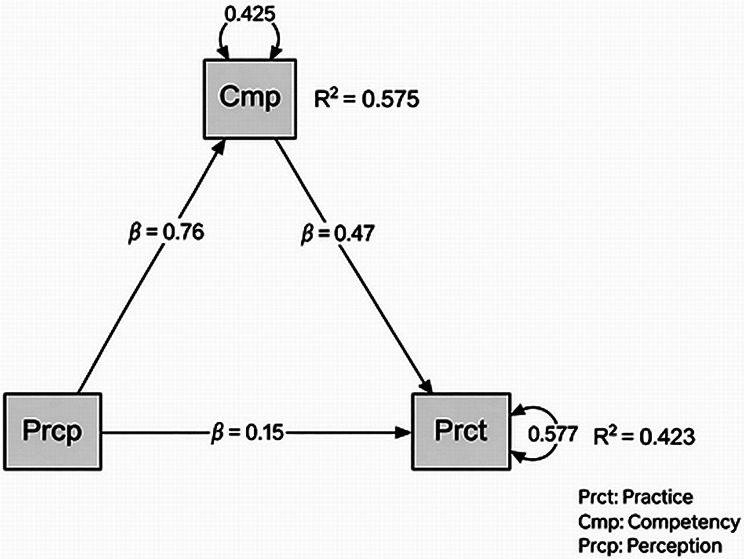
Path plot for the relationship between spiritual competency, spiritual perception, and practice. Prct” refers to Spiritual Care Practice

Figure 1 presents the standardized path coefficients of the structural equation model. The path from spiritual perception to spiritual competency was strong and statistically significant (β = 0.76, *p* < 0.001). Spiritual competency had a moderate positive effect on Spiritual Care Practice (β = 0.47, *p* < 0.001). The direct path from spiritual perception to Spiritual Care Practice was relatively weak but remained statistically significant (β = 0.15, *p* = 0.004).

Table 6 presents the mediation analysis results, showing direct, indirect, and total effects in the structural equation model. Spiritual competency partially mediated the relationship between spiritual perception and Spiritual Care Practice. The direct effect of spiritual perception on Spiritual Care Practice was statistically significant but small in magnitude (unstandardized β = 0.058, SE = 0.020, z = 2.918, *p* = 0.004; standardized β = 0.145). The effect of spiritual competency on Spiritual Care Practice was substantial (unstandardized β = 0.193, SE = 0.012, z = 16.626, *p* < 0.001; standardized β = 0.470). The indirect effect of spiritual perception on Spiritual Care Practice via spiritual competency was significant and substantial (unstandardized β = 0.302, SE = 0.025, z = 12.236, *p* < 0.001; standardized β = 0.357), indicating that spiritual competency plays a key mediating role and accounts for the majority of the total association. The total effect of spiritual perception on Spiritual Care Practice was moderate to strong (unstandardized β = 0.359, SE = 0.018, z = 19.512, *p* < 0.001; standardized β = 0.502).

**Table 6:** Analysis of spiritual competency mediation on the relationship between spiritual perception, engagement and barrier awareness

Effect Type	Path / Effect	Unstandardized Estimate	SE	z-value	*p*-value	Standardized β	95% CI
Direct Effects	Perception → Engagement and Barrier Awareness	0.058	0.020	2.918	0.004	0.145	0.019–0.096
	Competency → Engagement and Barrier Awareness	0.193	0.012	16.626	< 0.001	0.470	0.171–0.216
	Perception → Competency	1.560	0.086	18.074	< 0.001	0.758	1.391–1.729
Indirect Effect	Perception → Competency → Engagement and Barrier Awareness	0.302	0.025	12.236	< 0.001	0.357	0.253–0.350
Total Effect	Perception → Engagement and Barrier Awareness	0.359	0.018	19.512	< 0.001	0.502	0.323–0.395

### Moderation analysis of demographic variables

To examine whether the relationships among spiritual perception, spiritual competency, and Spiritual Care Practice varied by demographic characteristics, we conducted a moderation analysis using dummy-coded interactions for categorical variables. Categorical moderators were dummy-coded as follows: gender (Male = 0, Female = 1), education level (Bachelor’s = 0, Technical = 1, Master’s = 1), and ICU type (General ICU = 0, Medical ICU = 1, Surgical ICU = 1, Trauma ICU = 1). Coronary ICU and Neurological ICU were excluded from the analysis due to small sample sizes (*n* < 10). Moderation was tested by adding interaction terms (e.g., Spiritual Perception × ICU_Type_Surgical ICU) to the structural model and evaluating their significance. The results are presented in Table 7.

The moderation analysis revealed that education level and ICU type demonstrated significant interaction effects on selected structural pathways, while gender did not significantly moderate any path (all *p* > 0.05). Specifically, Master’s degree (vs. Bachelor’s) significantly moderated the paths from spiritual perception to Spiritual Care Practice (β = 0.436, *p* < 0.001) and from spiritual competency to Spiritual Care Practice (β = -0.251, *p* < 0.001). Additionally, Surgical ICU (vs. General ICU) significantly moderated the paths from spiritual perception to spiritual competency (β = -1.152, *p* < 0.001) and from spiritual perception to Spiritual Care Practice (β = -0.242, *p* < 0.001). These findings suggest that the relationships among spiritual perception, competency, and practice differ across education levels and ICU types, particularly for nurses with Master’s degrees and those working in Surgical ICUs.

No significant moderation effects were observed for gender or Trauma ICU (all *p* > 0.05). The wide confidence intervals for some ICU type moderators (e.g., Trauma ICU) suggest high variability or small sample sizes in these subgroups, and interpretations should be made cautiously. Due to the exploratory nature of this analysis, no correction for multiple testing was applied.

In Table 8, continuous moderators (age, years of ICU experience) were also analyzed, with age showing significant moderation effects on the paths from spiritual perception to Spiritual Care Practice (β = 0.017, *p* < 0.001) and from spiritual competency to Spiritual Care Practice (β = 0.012, *p* < 0.001). Years of ICU experience did not significantly moderate any path (all *p* > 0.05).

**Table 7:** Moderation analysis of demographic variables on structural paths using dummy-coded interactions

Path	Predictor	Moderator	Estimate (β)	SE	*p*-value	95% CI
**Education Level** (Reference: Bachelor’s = 0)						
Spiritual Care Practice	Spiritual Perception	Education_Technical	-0.029	0.035	0.405	[-0.097, 0.039]
Spiritual Care Practice	Spiritual Perception	Education_Masters	0.436	0.066	< 0.001	[0.306, 0.567]
Spiritual Competency	Spiritual Perception	Education_Technical	0.208	0.173	0.229	[-0.132, 0.549]
Spiritual Competency	Spiritual Perception	Education_Masters	-2.835	0.313	< 0.001	[-3.451, -2.219]
Spiritual Care Practice	Spiritual Competency	Education_Technical	0.044	0.045	0.337	[-0.046, 0.133]
Spiritual Care Practice	Spiritual Competency	Education_Masters	-0.251	0.033	< 0.001	[-0.316, -0.185]
**Gender** (Reference: Male = 0)						
Spiritual Care Practice	Spiritual Perception	Gender_Female	0.033	0.030	0.273	[-0.026, 0.092]
Spiritual Competency	Spiritual Perception	Gender_Female	-0.001	0.153	0.993	[-0.303, 0.301]
Spiritual Care Practice	Spiritual Competency	Gender_Female	0.001	0.029	0.983	[-0.057, 0.058]
**ICU Type** (Reference: General ICU = 0)						
Spiritual Care Practice	Spiritual Perception	ICU_Type_Medical ICU	0.149	0.157	0.347	[-0.166, 0.463]
Spiritual Care Practice	Spiritual Perception	ICU_Type_Surgical ICU	-0.242	0.054	< 0.001	[-0.350, -0.133]
Spiritual Care Practice	Spiritual Perception	ICU_Type_Trauma ICU	-0.559	1.809	0.758	[-4.183, 3.065]
Spiritual Competency	Spiritual Perception	ICU_Type_Medical ICU	1.099	0.700	0.122	[-0.303, 2.501]
Spiritual Competency	Spiritual Perception	ICU_Type_Surgical ICU	-1.152	0.226	< 0.001	[-1.605, -0.699]
Spiritual Competency	Spiritual Perception	ICU_Type_Trauma ICU	-2.482	8.124	0.761	[-18.757, 13.794]
Spiritual Care Practice	Spiritual Competency	ICU_Type_Medical ICU	-0.013	0.017	0.454	[-0.046, 0.021]
Spiritual Care Practice	Spiritual Competency	ICU_Type_Surgical ICU	-0.024	0.017	0.164	[-0.059, 0.010]
Spiritual Care Practice	Spiritual Competency	ICU_Type_Trauma ICU	-0.008	0.006	0.157	[-0.020, 0.003]

Notes: Reference Categories: Gender: Male = 0; Education Level: Bachelor’s = 0; ICU Type: General ICU = 0. Coronary ICU and Neurological ICU were excluded from the analysis due to small sample sizes (*n* < 10). All β values are unstandardized estimates

**Table 8:** Moderation analysis of continuous demographic variables

Path	Predictor	Moderator	Estimate (β)	SE	z	*p*-value	Standardized (β)	95% CI
Spiritual Care Practice	Spiritual Perception	Age	0.017	0.002	8.582	< 0.001	0.017	[0.013, 0.021]
Spiritual Care Practice	Spiritual Competency	Age	0.012	0.001	8.203	< 0.001	0.012	[0.010, 0.014]
Spiritual Care Practice	Spiritual Perception	Years of ICU Experience	0.002	0.003	0.617	0.537	0.002	[-0.004, 0.008]
Spiritual Care Practice	Spiritual Competency	Years of ICU Experience	0.002	0.002	0.669	0.504	0.002	[0.000, 0.004]
Spiritual Competency	Spiritual Perception	Age	0.003	0.001	1.761	0.078	0.003	[0.000, 0.005]
Spiritual Competency	Spiritual Perception	Years of ICU Experience	0.002	0.002	1.049	0.294	0.002	[-0.002, 0.006]

Note: All β values are unstandardized estimates

## Discussion

The current study aimed to examine the relationship between spiritual perception, competency, and practice among critical care nurses. Specifically, it investigated the mediating role of spiritual competency and the moderating role of demographic variables. We developed four research hypotheses according to this aim.

The first hypothesis was that there would be a positive relationship between spiritual perception and Spiritual Care Practice among critical care nurses, a finding supported in our study. This result aligns with previous studies that have emphasized the role of nurses’ perceptions of spirituality in their willingness to engage in spiritual care. Melhem et al., (2016) found that registered nurses with a strong understanding and appreciation of spirituality are more likely to incorporate spiritual care into their daily practice. This relationship may be attributed to nurses with heightened awareness of spiritual dimensions feeling more obligated or motivated to address these aspects in patient care, thereby enhancing their Spiritual Care Practice [20]. The Spiritual Care Practice construct in this study reflects nurses’ spiritual care engagement and their awareness of contextual barriers affecting spiritual care delivery.

In the present SEM model, the Spiritual Care Practice latent construct was operationalized as a higher-order factor that included both the frequency of spiritual support provided and nurses’ reported recognition of barriers to spiritual care. The positive loading of the barrier’s subscale should therefore be interpreted cautiously. One possible explanation is that nurses who report more frequent Spiritual Care Practice may also be more aware of the practical, organizational, and contextual barriers encountered during care delivery. However, this finding may also reflect the wording or structure of the measurement instrument, or the way respondents interpreted barrier-related items. Therefore, positive loading should not be interpreted as evidence that barriers enhance or promote Spiritual Care Practice. Rather, it suggests that barrier awareness was statistically associated with the broader practice construct in this model. Further psychometric evaluation is needed to clarify the meaning of this loading pattern among ICU nurses [21, 22]. 

We acknowledge that modeling perceived barriers as a positive reflective indicator of practice is unconventional. High barrier awareness may reflect organizational constraints (e.g., lack of time, privacy, or training) that suppress practice rather than indicate stronger practice. Our theoretical rationale, grounded in Watson’s Theory of Human Caring and reflective practice literature, posits that nurses who more actively engage in spiritual care also encounter and recognize more barriers, reflecting advanced professional consciousness. Nevertheless, this operationalization conflates practice enactment with contextual obstacle awareness, and findings related to Spiritual Care Practice should be interpreted with this caveat in mind. Future research should test alternative model specifications in which barriers are modeled as a separate exogenous contextual variable or as a distinct outcome to clarify their structural role.

However, the direct effect of spiritual perception on Spiritual Care Practice, though significant, was relatively less than expected. This suggests that while spiritual perception is essential, other mediating factors may influence how it translates into practical action. This finding aligns with previous studies of Baysal et al. (2024) and Birimoglu et al. (2024), who suggested that spiritual perceptions alone may not be sufficient to ensure comprehensive spiritual care without the competencies acquired through education and training among nursing students [22, 23]. 

This supports the second hypothesis, which suggests that spiritual competency mediates the relationship between spiritual perception and Spiritual Care Practice. Also, the findings of the current study strongly support this hypothesis. The mediation analysis revealed that spiritual competency substantially enhances the impact of spiritual perception on spiritual practice. This aligns with Bandura’s Self-Efficacy Theory, which posits that individuals are more likely to engage in a behavior if they believe they can execute it successfully [5]. In this context, critical care nurses who perceive spirituality as necessary and simultaneously feel competent in delivering spiritual care are more likely to put their spiritual perceptions into Spiritual Care Practice.

The convincing mediator observed in the current study suggests that competency development is a critical lever for improving nurses’ Spiritual Care Practice s. This finding is supported by Wang et al., (2024), who highlighted the importance of spiritual care education in enhancing nurses’ confidence and skills, thereby increasing the frequency and quality of Spiritual Care Practice s [24]. 

The results partially supported Hypothesis 3. The moderation findings were pathway-specific. Age, ICU type, and education level demonstrated significant interaction effects on selected relationships, whereas gender and years of ICU experience did not consistently influence the examined pathways. Hypothesis 4 was also partially supported. Gender did not significantly moderate any path, while age, ICU type, and education level moderated selected structural pathways. Years of ICU experience did not significantly moderate any examined relationship. Gender did not significantly moderate any path in this study. This result does not align with previous studies across various cultural contexts, which have consistently shown that female nurses tend to report higher spiritual sensitivity, greater engagement with spiritual care concepts, and stronger links between spiritual perception and competency than their male counterparts. Such gender differences may reflect socialization patterns, differing caring orientations, or varying levels of comfort in addressing existential and spiritual issues. Notably, while gender did not significantly moderate the direct pathway from spiritual perception to Spiritual Care Practice (Hypothesis 3), it did significantly moderate the pathway from spiritual perception to spiritual competency (Hypothesis 4) [25–27].

The moderation effect of age suggests that older nurses may be better equipped to translate spiritual perceptions and competencies into Spiritual Care Practice. This finding may reflect cumulative professional experience and, over time, greater exposure to diverse patient needs, which enhances their ability to integrate spiritual care into their Spiritual Care Practice. This finding is consistent with Gupta & Chadha (2013), who found that age is positively associated with both perceptions of spiritual care and its practice among individuals [27]. 

The significant moderation by ICU type (e.g., Surgical ICU vs. General ICU) suggests that the relationships among spiritual perception, spiritual competency, and Spiritual Care Practice differ across critical care settings.

In such contexts, nurses’ spiritual perception may be more frequently invoked to meet patients’ and their families’ spiritual needs, and spiritual competency may be more directly translated into observable Spiritual Care Practice. One possible explanation is the stronger associations observed in these units. Prolonged nurse–patient interaction may strengthen the pathway from spiritual perception to spiritual competency and, subsequently, to Spiritual Care Practice. The nature of work in this ICU environment requires rapid clinical workflows for rapid stabilization and standardized protocols [28, 29]. 

These findings may be considered within the broader cultural context of Egypt, where religious and spiritual values may influence perceptions of illness and caregiving. However, cultural or religious influences were not directly measured in this study and should therefore be interpreted cautiously. In such contexts, spiritual perception may be particularly salient because religion and spirituality are often not sharply differentiated, fostering a holistic view of care that integrates existential and religious dimensions. Similar cultural dynamics appear in other regional studies [28, 29]. 

### Limitation

Despite applying SEM for mediation and moderation in this study, we were constrained in the number of moderator variables we could include to reduce model complexity and achieve model fitness. Also, several indicators showed very high factor loadings (> 0.95), indicating strong convergent validity but also possible conceptual overlap between some subdimensions of spiritual perception and competency. This may limit the discriminant clarity of the constructs and should be addressed in future measurement refinement.

Although SEM was used to examine associations among spiritual perception, competency, and practice, the just-identified structural model limits the interpretation of global fit indices for evaluating the structural relationships. Therefore, the reported pathways should be interpreted as statistically modeled associations rather than confirmation of causal mechanisms.

A measurement consideration is that the Spiritual Care Practice construct incorporated both spiritual support behaviors and perceived barriers to care. While barrier awareness may reflect nurses’ active engagement with spiritual care challenges, barriers may also represent organizational constraints that limit care delivery. Future research should examine alternative measurement models in which barriers are analyzed as an independent contextual factor. Although barrier awareness may reflect nurses’ active engagement with spiritual care challenges, barriers may also represent organizational constraints that limit care delivery, such as workload, limited time, privacy concerns, or staffing issues. Therefore, future research should examine alternative measurement models in which barriers are analyzed as an independent contextual factor.

The internal consistency of the “Barriers to providing spiritual care” subscale was modest in the original development study (Cronbach’s α = 0.64; Gallison et al., 2013). In the current Egyptian sample, reliability was acceptable (Cronbach’s α = 0.82). Since this subscale contributes to the broader “Spiritual Care Practice " construct, its conceptual heterogeneity in the original study should be noted, though this was less problematic in our sample. Future studies should aim to improve psychometric properties to enhance reliability.

All instruments were administered in their original English versions. Although the nursing workforce in Egypt typically receives English-language education, English-only instruments may have affected comprehension and cultural validity for some participants. Future research should consider translated and culturally adapted instruments to enhance semantic and conceptual equivalence.

Finally, as the data were self-reported, social desirability bias may be a concern. This is especially important in spirituality-related research, where respondents may provide socially desirable answers due to the sensitive and value-laden nature of spiritual and religious topics. Although anonymity and confidentiality were assured to encourage honest responses, the influence of socially desirable responding cannot be completely ruled out.

A key limitation is reliance on a single-wave, self-report design, which raises the possibility that common method variance will inflate associations among variables. Cross-sectional SEM cannot establish causal or temporal mediation. This concern is particularly relevant for spirituality measures, where respondents may provide socially desirable answers. As a result, the magnitude of the reported relationships may be overestimated. Future research could mitigate this issue by using multi-source or longitudinal data, behavioral measures, or a social desirability scale.

This study’s cross-sectional design precludes any causal inference regarding the observed associations. Online recruitment may have excluded nurses with less digital access or lower engagement, potentially introducing selection bias toward younger, more digitally literate, or more motivated nurses. The use of convenience sampling and online recruitment may limit the representativeness of the sample and introduce selection bias.

### Implications for nursing and health policy

This study yields several evidence-based implications for nursing practice and health policy that are directly tied to the findings. First, the strong mediating role of spiritual care competency (standardized indirect effect β = 0.357) indicates that competency development is the critical lever for translating spiritual perception into practice. Healthcare institutions and nursing education programs should develop systematic spiritual care competency training for ICU nurses, with explicit focus on the subdimensions that demonstrated the highest relative weights: communication (weight = 0.364), assessment and implementation (weight = 0.170), and referral to professionals (weight = 0.185). Training curricula should include communication skills, spiritual assessment protocols, and appropriate referral pathways to chaplaincy or religious professionals, where culturally appropriate, particularly in the Egyptian context, where spiritual traditions are central to patient coping.

Second, the significant moderating effects of age and ICU type suggest that one-size-fits-all training is insufficient. Tailored training should be developed for nurses in Surgical ICUs, where the relationships among perception, competency, and Spiritual Care Practice were attenuated compared with General ICUs. Mentorship programs pairing younger or specialized-ICU nurses with more experienced colleagues may help bridge these competency-to-practice gaps.

Third, ICU-specific spiritual care protocols should be created to address the contextual barriers identified in this study. Because the Barriers subscale loaded positively onto the practice construct—suggesting that active spiritual care engagement co-occurs with awareness of obstacles—hospital administrators must concurrently address organizational barriers such as time constraints, lack of privacy, inadequate staffing, and limited training resources. Without structural support, competency gains may not translate into sustained practice.

Fourth, given that spiritual care competency explained 57.5% of the variance in the model, integrating spiritual care education into critical care nursing curricula and continuing professional development should be a policy priority. Accreditation bodies and hospital administrators should mandate spiritual care competencies—specifically assessment, communication, personal support, and referral—as core competencies for ICU nursing certification, rather than optional adjuncts to technical training.

## Conclusion

The results of this study provide important insights into how critical care nurses’ perceptions of spirituality, their competency in spiritual care, and their practice of spiritual care are interconnected. These findings indicate a connection among spiritual care competency, spiritual perceptions, and Spiritual Care Practice, suggesting that competency may influence how perceptions relate to Spiritual Care Practice, though causality cannot be determined. Also, our findings should be viewed considering the study’s cross-sectional design, which precludes causal conclusions, and the use of self-reported measures that may be biased and subject to measurement error. Results indicate associations, not definitive relationships, and should be confirmed in future longitudinal and robust studies. The mediation association suggests that tailored interventions might be needed to address the specific needs of different demographic groups or those working in specialized ICUs.

## Data Availability

For scientific studies, the corresponding researcher will provide information suitable for research.
